# Prenatal exposure to bisphenol A and/or diethylhexyl phthalate alters stress responses in rat offspring in a sex- and dose-dependent manner

**DOI:** 10.3389/ftox.2023.1264238

**Published:** 2023-12-13

**Authors:** Amrita Kaimal, Jessica M. Hooversmith, Ariana D. Cherry, Jillian T. Garrity, Maryam H. Al Mansi, Nicholas M. Martin, Hannah Buechter, Philip V. Holmes, Puliyur S. MohanKumar, Sheba M. J. MohanKumar

**Affiliations:** ^1^ Neuroendocrine Research Laboratory, University of Georgia, Athens, GA, United States; ^2^ Biomedical and Translational Sciences Institute, Neuroscience Division, University of Georgia, Athens, GA, United States; ^3^ Behavioral Neuropharmacology Laboratory, University of Georgia, Athens, GA, United States

**Keywords:** BPA, DEHP, mixture, prenatal exposure, behavior, sex differences

## Abstract

**Background:** Prenatal exposures to endocrine disrupting chemicals (EDCs) are correlated with adverse behavioral outcomes, but the effects of combinations of these chemicals are unclear. The aim of this study was to determine the dose-dependent effects of prenatal exposure to EDCs on male and female behavior.

**Methods:** Pregnant Sprague-Dawley rats were orally dosed with vehicle, bisphenol A (BPA) (5 μg/kg body weight (BW)/day), low-dose (LD) diethylhexyl phthalate (DEHP) (5 μg/kg BW/day), high-dose (HD) DEHP (7.5 mg/kg BW/day), a combination of BPA and LD-DEHP (B + D (LD)), or a combination of BPA and HD-DEHP (B + D (HD)) on gestational days 6–21. Adult offspring were subjected to the Open Field Test (OFT), Elevated Plus Maze (EPM), and Shock Probe Defensive Burying test (SPDB) in adulthood. Body, adrenal gland, and pituitary gland weights were collected at sacrifice. Corticosterone (CORT) was measured in the serum.

**Results:** Female EDC-exposed offspring showed anxiolytic effects in the OFT, while male offspring were unaffected. DEHP (HD) male offspring demonstrated a feminization of behavior in the EPM. Most EDC-exposed male offspring buried less in the SPDB, while their female counterparts showed reduced shock reactivity, indicating sex-specific maladaptive alterations in defensive behaviors. Additionally, DEHP (LD) males and females and B + D (LD) females displayed increased immobility in this test. DEHP (LD) alone and in combination with BPA led to lower adrenal gland weights, but only in male offspring. Finally, females treated with a mixture of B + D (HD) had elevated CORT levels.

**Conclusion:** Prenatal exposure to BPA, DEHP, or a mixture of the two, affects behavior, CORT levels, and adrenal gland weights in a sex- and dose-dependent manner.

## 1 Introduction

The global rise in the prevalence of neuropsychiatric disorders can be partially attributed to the widespread use of endocrine disrupting chemicals (EDCs) (Kajta and Wójtowicz, 2013; Mustieles et al., 2015). These environmental contaminants have been repeatedly associated with reproductive (Sifakis et al., 2017), metabolic (Janardhanan, 2018), and developmental (Mouritsen et al., 2010) abnormalities. Some of the most prevalent EDCs in the environment are the plasticizers bisphenol A (BPA) and diethylhexyl phthalate (DEHP), which are typically found in water bottles and food can linings (Chapin et al., 2008), medical devices (Erythropel et al., 2014), and personal care products (Rowdhwal and Chen, 2018). Humans are primarily exposed to these particular EDCs via ingestion of contaminated food and beverages containing BPA and/or DEHP that has leached from the plastics (Kang et al., 2006; Erythropel et al., 2014).

In pregnant females, these chemicals can readily cross the placental barrier and affect fetal brain development *in utero* (Singh et al., 1975; Balakrishnan et al., 2010). Additionally, there appears to be a sex bias in the prevalence of multiple neuropsychiatric disorders (Ramtekkar et al., 2010; Altemus et al., 2014; Baio et al., 2018). It is highly likely that sex differences in the risk for developing these disorders may stem from exposures to EDCs *in utero*, a sensitive critical period of fetal development (Mallozzi et al., 2016).

Exposure to BPA during the *in utero* and early postnatal stages exerts a variety of sex-specific effects on stress-related behaviors and hormones, including corticosterone (CORT). Low-dose prenatal BPA exposure (2–200 μg/kg/day) induces anxiogenic effects and reduces locomotor activity in the Open Field Test (OFT) in female mouse offspring; however, the reverse is observed in males (Kundakovic et al., 2013). These effects are compounded with increasing doses, suggesting a dose-dependent response. Furthermore, perinatal treatment with 5 μg/kg of BPA eliminates sex differences in rats in the Elevated Plus Maze (EPM), without affecting learning and memory in the Morris Water Maze (Jones and Watson, 2012). In addition, studies report increased basal and post-stress levels of CORT accompanied by increased anxiety-like behavior in female rat offspring with perinatal BPA exposure (40 μg/kg/day) (Poimenova et al., 2010). BPA-exposed male offspring, on the other hand, display an even higher post-stress CORT response than females. However, perinatal treatment with a lower BPA dose (2 μg/kg/day) can induce anxiolytic effects in female rat offspring without affecting CORT levels before or after stress exposure (Chen et al., 2014). Yet their BPA-exposed male counterparts have persistently heightened pre- and post-stress CORT concentrations. The variability observed in BPA effects across studies may be attributed to methodological differences, including window and route of BPA exposure, the species studied, and age of offspring at evaluation.

Perinatal DEHP appears to have a non-monotonic dose response on exploratory and locomotor activity. Low DEHP doses (5–400 μg/kg/day) are associated with increased home cage exploration in mouse offspring of both sexes (Quinnies et al., 2017), whereas higher doses (10–200 mg/kg/day) lead to decreased locomotor activity in the OFT in female mice (Xu et al., 2015). Anxiogenic effects in the OFT and EPM are additionally observed in both male and female mice following low-dose (5 and 40 μg/kg) (Quinnies et al., 2017), as well as high-dose (50–750 mg/kg) (Dai et al., 2015; Xu et al., 2015; Barakat et al., 2018) perinatal DEHP exposure. Moreover, male offspring appear to show increased dose-dependent deficits in learning and memory following prenatal DEHP exposure (Lin et al., 2015; Barakat et al., 2018). Although no changes were observed in CORT in male offspring with high-dose DEHP exposure (Martinez-Arguelles et al., 2011), prenatal treatment with 150 mg/kg of DEHP has been shown to transgenerationally reduce CORT concentrations in female mouse offspring instead (Quinnies et al., 2015). Thus, it appears that alterations in CORT levels in EDC-exposed offspring depend on the type of EDC and dose used.

An especially problematic aspect of these contaminants is that, in reality, they exist in combination with one another in the environment–as mixtures. Determining the mechanisms of action underlying EDC mixtures is a relatively novel area of research, but studies agree that EDC mixture effects are more elusive and vary from those of individual EDCs (Biemann et al., 2014; Suteau et al., 2020). EDC mixtures, including BPA and DEHP combinations, are associated with a variety of consequences on the metabolic (Naville et al., 2013; Labaronne et al., 2017), reproductive (Borman et al., 2017; Balci et al., 2020; Christiansen et al., 2020), and cardiovascular (Shang et al., 2019) systems in male and female rodents. Yet, there is an alarming lack of data on sex-specific outcomes on behavior following exposures to BPA and DEHP mixtures.

The objective of the present study was to assess sex differences in stress-related behaviors, cognition, and the hypothalamic-pituitary-adrenal (HPA) axis following prenatal BPA or DEHP exposure individually or in combination at low and high doses. Our hypothesis was that prenatal EDC exposure, particularly in combination, would induce sex-specific and dose-dependent alterations in behavioral responses to stress that are mediated by the HPA axis.

## 2 Materials and methods

### 2.1 Animals

Adult female Sprague-Dawley (SD) rats were purchased from Envigo (Indianapolis, IN) and housed in rooms that were light- (12:12 light-dark cycle) and temperature-controlled (23.2°C ± 2°C, 50% ± 20% relative humidity) at the University of Georgia. Food and water were provided *ad libitum*. The rats were fed Pico Lab Rodent Diet 20 (LabDiet). Animals were housed in polycarbonate cages with corn cob bedding. Bisphenol exposures from the environment (cages, water bottles, etc.) were not controlled for since all animals were maintained in the same environment. After a week of acclimation, each of the female breeders underwent vaginal cytology for 10 consecutive days to track their individual estrous cycles. Once in proestrus, a female was randomly assigned a male by generating random numbers using the standard = RAND () function in Microsoft Excel, and the two were co-housed for 1 day. The presence of a vaginal plug was used to confirm the occurrence of mating. Gestational day (GD) 0 represented the day of copulation.

### 2.2 Chemicals

BPA (Lot MKBH 2096V; Catalog No. 239658; Purity: ≥99.0%) and DEHP (Lot BCBR8079V; Catalog No. 36735; Purity: ≥98.0%) were purchased from Sigma Aldrich (St. Louis, MO). Stock solutions were made in dimethylsulfoxide (DMSO; 1 μg/μL for BPA and low dose DEHP and 1 mg/μL for high dose DEHP). Doses were calculated daily based on BW and mixed with 20 µL Phosphate Buffered Saline (PBS) for oral dosing. The final concentration of DMSO in the daily dose was less than 10%. Daily oral dosing occurred from GD 6–21 as per our previously published study (Dagher et al., 2021). The vehicle (20 µL of PBS) or EDC treatments were discharged into the oral cavity using a micropipette to avoid any local irritation to the gastrointestinal tract and potential stress to the pregnant dam.

Since BPA has been studied extensively, we used it as a positive control and therefore only tested the effects of a single low dose. The BPA dose was selected because it is significantly lower than the Environmental Protection Agency (EPA) recommended no-observed-adverse-effect-level (NOAEL) dose of 5 mg/kg/day (EPA, 2010) and it is also 10-fold below the current daily reference dose of 50 μg/kg/day (Almeida et al., 2018). Additionally, this dose is within the estimated range of BPA exposure in humans (0.4–5 μg/kg/day) (Lakind and Naiman, 2008). The high dose of DEHP was selected since it is higher than the established NOAEL dose of 4.8 mg/kg/day (Blystone et al., 2010), whereas the low DEHP dose is significantly lower than this. The low dose of DEHP used in this study lies within the range of the typical daily intake of this chemical in adult humans (1–30 μg/kg/day) (Shelby, 2006), but is well below the EPA reference dose of 20 μg/kg/day (EPA, 1987).

### 2.3 Experimental design

**FIGURE 1:**
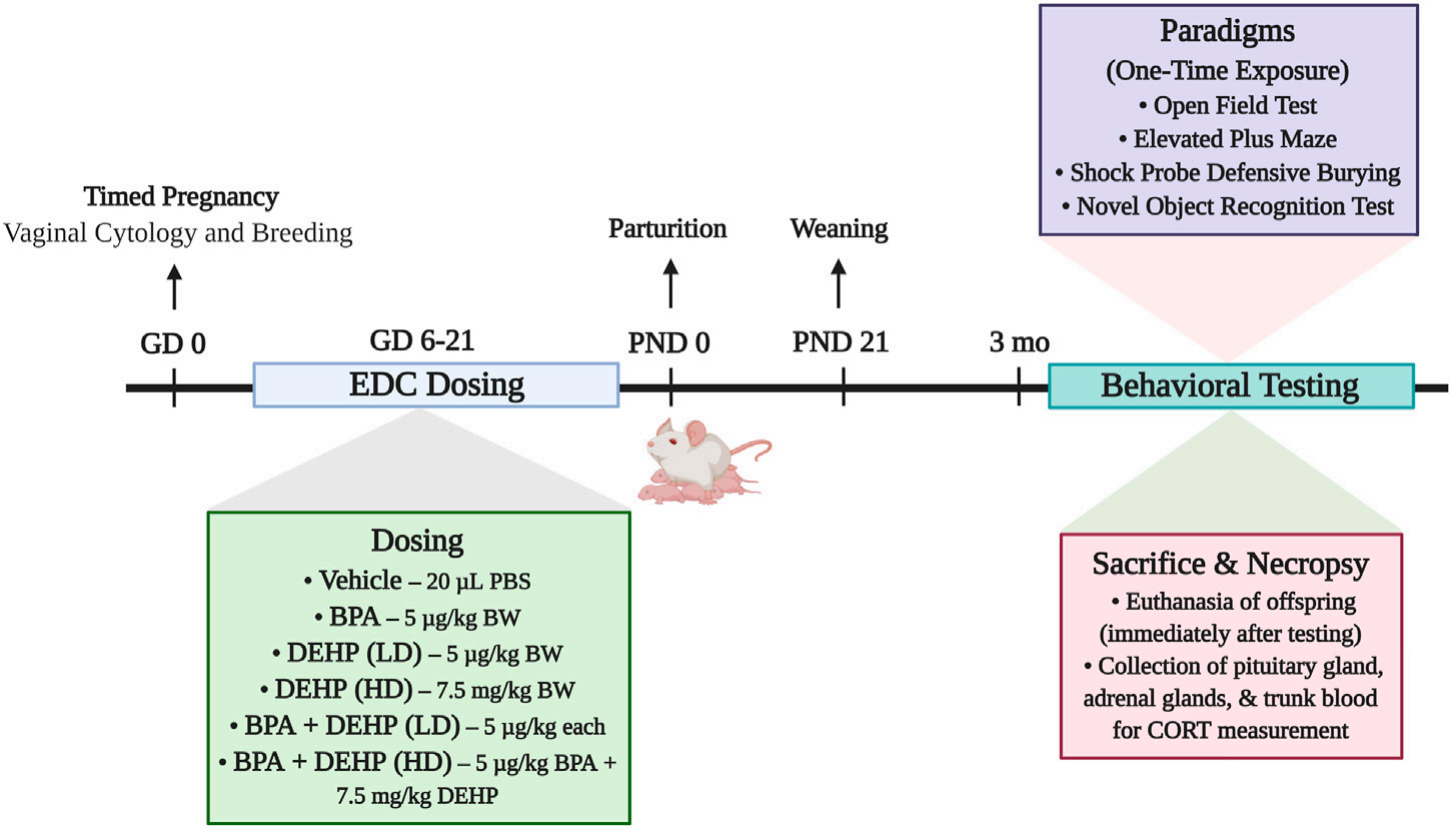
Summary of the experimental design of the study. Pregnant Sprague-Dawley dams were orally dosed daily from gestational days (GD) 6–21 with vehicle (Control) (20 µL PBS; *n* = 6), BPA (5 μg/kg/day; *n* = 7), low-dose (LD) DEHP (5 μg/kg/day; *n* = 6), high-dose (HD) DEHP (7.5 mg/kg/day; *n* = 6), a mixture of BPA + LD DEHP (5 μg/kg/day of BPA +5 μg/kg/day of DEHP; *n* = 6), or a mixture of BPA + HD DEHP (5 μg/kg/day of BPA +7.5 mg/kg/day of DEHP; *n* = 7). Adult male and female offspring aged 3 months or older were administered a battery of behavioral tests once, following which they were immediately euthanized. Pituitary and adrenal glands were dissected and weighed upon euthanasia. Trunk blood was collected for the measurement of serum CORT using radioimmunoassay. Experimental design schematic was created using Biorender. com. Note: EDC, endocrine disrupting chemical; PBS, Phosphate Buffered Saline; BPA, Bisphenol A; DEHP, diethylhexyl phthalate; LD, low-dose; HD, high-dose; BW, body weight; CORT, corticosterone.

The experimental design is demonstrated in Figure 1. The dam was considered the experimental unit. Each dam was randomly assigned to one of 6 different treatment groups: control (20 µL PBS; *n* = 6), BPA (5 μg/kg BW/day; *n* = 7), low-dose (LD) DEHP (5 μg/kg BW/day; *n* = 6), high-dose (HD) DEHP (7.5 mg/kg BW/day; *n* = 6), a combination of BPA and LD DEHP (5 μg/kg/day of BPA +5 μg/kg/day of DEHP; *n* = 6), and a combination of BPA and HD DEHP (5 μg/kg/day of BPA +7.5 mg/kg/day of DEHP; *n* = 7). Generation of random assignment numbers was completed using the standard = RAND () function in Microsoft Excel. Litters were equalized in number prior to weaning. No differences in developmental trajectory were observed in the mothers and offspring (Dagher et al., 2021). One male pup and one female pup from each dam then underwent behavioral testing when they were 3–4 months of age. This was immediately followed by euthanasia and collection of trunk blood and certain organs for further processing.

### 2.4 Behavioral testing

The adult male and female offspring of the treated dams were transferred to another facility on campus 1 week prior to behavioral testing, where they remained undisturbed during this habituation period for a week. Animals were group housed (two to four rats per cage) with rats of the same sex and dose group in polycarbonate cages with corn cob bedding. Rooms were maintained at 23.3°C ± 3°C on a 12:12 reverse light-dark cycle. All behavioral testing occurred during the dark cycle. All animals had access to food and water *ad libitum* in their home cages, including before and after each testing session.

The animals were administered a battery of behavioral tests including the Open Field Test (OFT), Elevated Plus Maze (EPM), and Shock Probe Defensive Burying (SPDB). The Novel Object Recognition test (NOR) was also administered, but only to the animals in the LD group since these offspring demonstrated more intriguing behavioral effects and we wanted to examine how their cognition was affected as a result. The order for the tests was OFT, EPM, SPDB, followed by NOR. The tests were administered in succession and each rat was exposed to each test only once. Rats were then euthanized after completion of the behavioral testing. Both testing and euthanasia for each rat occurred in the same day.

The animals’ behaviors in each test were video recorded by a direct overhead webcam (Microsoft), and all videos were manually scored by experimenters unaware of the treatment groups. Rats were habituated to the testing areas for 5 min prior to testing. They remained in their cages during this time. All boxes and equipment were disinfected between every trial. Vaginal smears were obtained from all female rats for 2–10 days prior to behavioral testing to ensure that females were tested when they were in estrus.

#### 2.4.1 Open field test (OFT)

Each animal was placed in a transparent plexiglass test chamber (43.3 cm long x 43.3 cm wide x 30.5 cm high) consisting of a center zone and a perimeter zone (Hooversmith et al., 2019). The area considered the center zone measured 25.3 cm^2^. At the beginning of the testing session, all rats were placed in the lower left corner of the box facing the opposite wall. The animal was allowed to freely explore the box for 10 min (Hooversmith et al., 2019). OFT behaviors were automatically recorded using Activity Monitor software (Med Associates, Fairfax, VT, United States) on a desktop computer to automate behavioral testing and provide unbiased analyses of data. The following measures were recorded during the testing session: number of entries and time spent in the center and perimeter zones, frequency and time spent rearing, as well as distance traveled, and time spent ambulating within the box. Animals were tested in the OFT under red lights.

#### 2.4.2 Elevated plus maze (EPM)

The testing apparatus consisted of a wooden maze painted black matte with two pairs of arms set perpendicular to each other and placed 50 cm above the floor. The arms extended from a central platform (9 cm^2^) and formed a pair of open arms (45 × 9 cm) and a pair of closed arms (45 × 9 × 38 cm) (Simone et al., 2015). The open arms were not bound by walls and the closed arms were enclosed by high walls and no ceilings. Animals were tested under red lights. To begin the testing session, the animal was placed on the central platform facing an open arm opposite the experimenter. The number of entries and time spent in each arm and crosses through the central platform were recorded during the 5-min testing session (Sciolino et al., 2012; Balasubramanian et al., 2014). Entry into an arm was considered as the presence of all four feet of the rat in the arm.

#### 2.4.3 Shock probe defensive burying (SPDB)

Animals were placed in a covered clear polycarbonate cage (20 × 40 × 20 cm) containing bedding at the beginning of the testing session. An electrified probe extended 6 cm into the cage and 2 cm above the bedding (Simone et al., 2015). The experimenter administered a mild shock of 3 mA DC (E13-08, Coulbourn Instruments, Allentown, PA) to the animal after initial contact with the probe. The intensity of the animal’s response to the shock, or shock reactivity, on a scale of one to four was manually recorded: 1—Flinch involving only head or forepaw, without immediate ambulation away from the probe; 2—Whole-body flinch and ambulation to far end of chamber; 3—Hopping away and running; 4—Jumping away and running. Following this, the frequency and time spent engaging in the following behaviors were measured during the 10-min testing session: burying, immobility, rearing, exploring the shock-probe, and grooming. Testing occurred under red lights. After returning the rat to its home cage at the end of the session, the height of the highest point of the bedding (representing the ending height) was measured and manually recorded.

#### 2.4.4 Novel object recognition test (NOR)

The test chambers had opaque walls with no ceiling (52 × 35 × 32.5 cm, Sterlite). A variety of objects that varied in size (maximum size: 10.5 cm high x 18.5 cm wide), shape, and material (plastic, glass, metal, and ceramic) were used. These particular objects were selected because they were previously tested for object preference bias by Simone et al. (Simone et al., 2015), and no significant differences were found in bias or exploration of these objects. All objects were glued to a small jar that was fixed in place by screwing the jar to a lid within the box. The test consisted of the following procedure: 1) The rat was allowed to freely explore two randomized identical objects for 5 min during the familiarization phase (T1). Randomization was completed using a computer based random number generator. 2) The rat was returned to its home cage with food and water for 45 min during the retention phase. 3) The rat was returned to the test chamber for 3 min for the test phase (T2), in which one of the identical objects from T1 was replaced with a novel object. The other identical T1 object was replaced with a duplicate object. The novel object and locations of the objects in T2 were counterbalanced across all rats. Animals were tested in the NOR under red lights.

For the T1 phase, the average exploration time was measured, and the discrimination index (DI) was calculated. Exploration was defined as sniffing the object within 2 cm from the edge or touching the object. Climbing on/over or rearing on the objects were not counted as part of the exploration time. DI in this phase was defined as the difference in exploration time between the right object and left object divided by the total exploration time of the right and left objects (Aubele et al., 2008). For the T2 phase, we calculated both the DI and the recognition index (RI). DI in this phase was defined as the difference in exploration time between the novel object and familiar object divided by the total exploration time of the novel and familiar objects (Ennaceur and Delacour, 1988). Finally, the RI was considered the main index of novel object recognition in our study because it is a more sensitive measure of recognition memory compared to the DI, which may be influenced by differences in exploration levels across animals (Akkerman et al., 2012; Antunes and Biala, 2012). The RI was defined as the percent of time spent exploring the novel object relative to the total amount of time spent exploring both the novel and familiar objects (Mumby et al., 2002; Botton et al., 2010).

### 2.5 Tissue collection and preparation

Immediately following behavioral testing, female offspring in estrus (as confirmed by vaginal cytology) and male offspring were euthanized by rapid decapitation. Pituitary glands and adrenal glands were dissected, weighed, and stored at −80°C for further processing.

### 2.6 Corticosterone measurement

Following the sacrifice of the rats, trunk blood was collected, centrifuged, and serum was separated and stored at −80°C. Serum corticosterone levels were measured in duplicate using a double antibody radioimmunoassay (MP Biomedicals, Santa Ana, CA; SKU:07120121), according to the manufacturer’s protocol. Values were expressed as ng/mL.

### 2.7 Statistical analysis

Prism 9.0.0 (GraphPad, Inc.) software was used to perform statistical analyses. Behavioral data, organ weights, and corticosterone levels were analyzed by two-way ANOVA (EDC exposure × sex). Interaction effects between EDC exposure and sex were also assessed. One-way ANOVA analyses were further used to identify main effects of EDC exposure separately by sex. Statistical differences between control and EDC groups in behavioral parameters were measured using Fisher’s LSD *post hoc* test. Differences between control and EDC groups in body weights, organ weights, and corticosterone levels were analyzed using Tukey’s multiple comparisons *post hoc* test. Values that were statistical outliers were excluded from all of the analyses. *p*-value <0.05 was considered to indicate a statistically significant difference. Data was expressed as mean ± standard error of mean (SEM).

## 3 Results

### 3.1 Open field test

**FIGURE 2:**
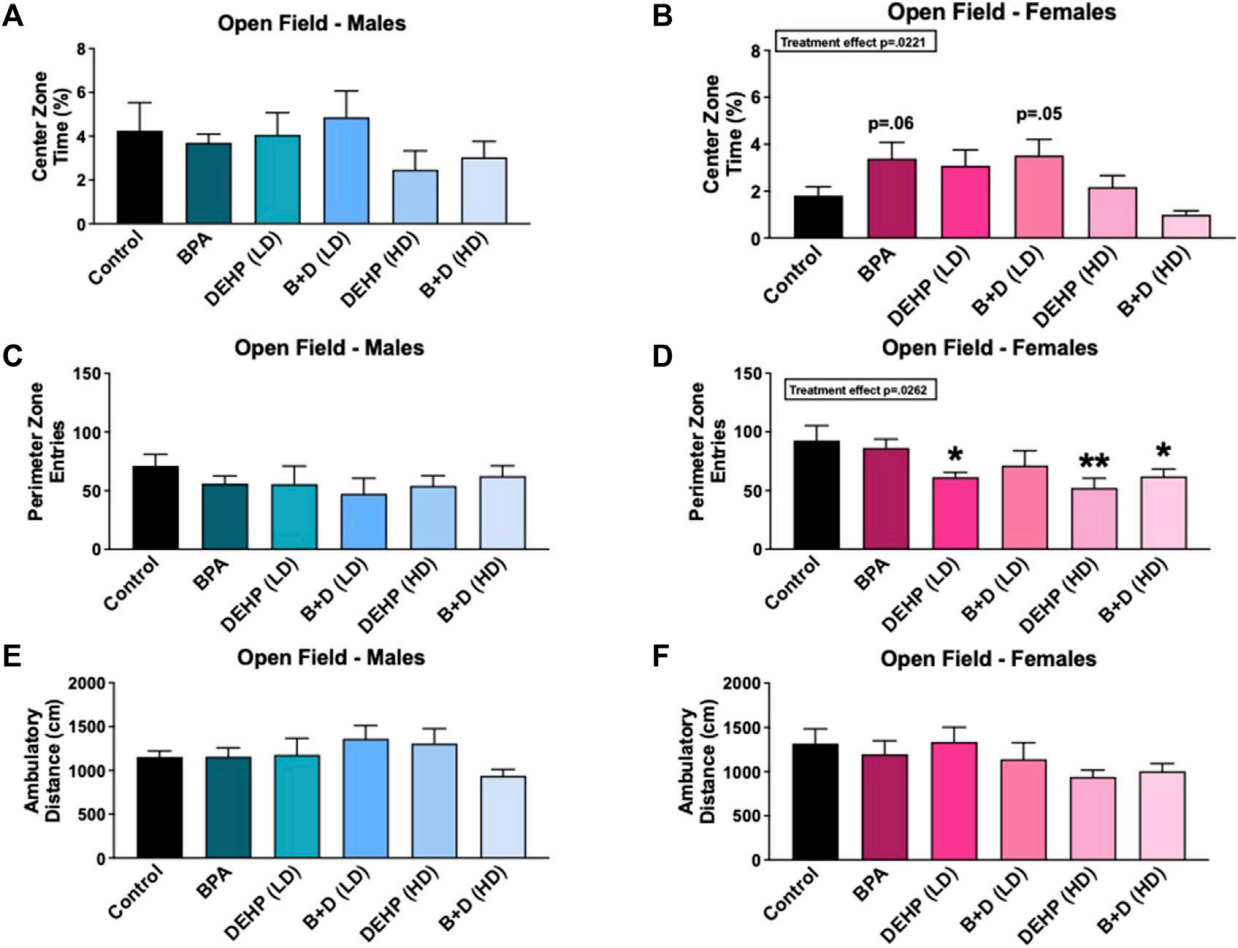
Behavioral effects of prenatal exposure to vehicle or EDCs in female and male rat offspring in the open field test (OFT). **(A)** Center zone time in female offspring, **(B)** center zone time in male offspring, **(C)** perimeter zone entries in female offspring, **(D)** perimeter zone entries in male offspring, **(E)** locomotor activity in female offspring, and **(F)** locomotor activity in male offspring. Behavioral data were collected from adult male and female offspring prenatally exposed to vehicle (Control) (males: *n* = 6; females: *n* = 5–6), BPA (males: *n* = 6; females: *n* = 7), DEHP (LD) (males: *n* = 5–6; females: *n* = 6), DEHP (HD) (males: *n* = 5–6; females: *n* = 6), a mixture of BPA + DEHP (LD) (males: *n* = 5; females: *n* = 6), or a mixture of BPA + DEHP (HD) (males: *n* = 7; females: *n* = 6). Data were analyzed by one-way ANOVA, followed by Fisher’s LSD *post hoc* test. ^*^ *p* < 0.05, ^**^ *p* < 0.01, comparison between control and EDC-exposed female offspring. Error bars represent the standard error of the mean (SEM).

No significant differences were observed in distance traveled (Figures 2E,F) or time spent (Table 1) ambulating within the chamber in males or females from any of the groups. A significant main effect of sex (F (1, 58) = 8.4, *p* = 0.0054) was observed in rearing frequency (mean ± SEM) (Table 1). DEHP (HD) females (114.0 ± 29.7) displayed a marked increase in rearing compared to their male counterparts (53.2 ± 2.7; *p* = 0.002), suggesting a possible anxiety-like response in DEHP (HD) females. No additional differences were found in this measure, or in rearing time (Table 1).

**TABLE 1:** Behavioral data of male and female offspring following low-dose (5 µg) and high-dose (7.5 mg) prenatal EDC exposure.

Behavioral parameter	Control		BPA (5 µg)		DEHP (5 µg)		BPA ^a^5 µg DEHP		DEHP (7.5 mg)		BPA ^a^7.5 mg DEHP		Sex effect	
	Male	Female	Male	Female	Male	Female	Male	Female	Male	Female	Male	Female		
OFT														
Ambulation (% time)	19.9 ± 1.8	21.6 ± 2.8	21.7 ± 1.6	17.6 ± 2.4	19.3 ± 3.2	22.5 ± 2.7	20.7 ± 2.1	18.2 ± 3.4	23.1 ± 3.4	15.0 ± 1.3	17.7 ± 1.8	16.4 ± 1.6	NS	
Rearing (frequency)	69.3 ± 10.2	82.7 ± 14.9	67.3 ± 14.7	69.2 ± 1.4	69.2 ± 7.7	80.2 ± 4.7	71.6 ± 11.2	91.7 ± 10.7	53.2 ± 2.7 **^b^**	114.0 ± 29.7 **^b^**	60.9 ± 8.8	83.2 ± 6.6	*p* = .0054	
Rearing (% time)	21.7 ± 3.4	21.6 ± 4.4	16.5 ± 3.0	25.6 ± 4.7	20.0 ± 2.9	24.0 ± 2.7	18.7 ± 3.6	24.7 ± 2.5	18.7 ± 1.6	23.1 ± 2.2	18.3 ± 4.1	16.4 ± 1.4	NS	
Center (% time)	4.3 ± 1.3 **^a^**	1.8 ± 0.4 **^a^**	3.7 ± 0.4	3.4 ± 0.7	4.1 ± 1.0	3.1 ± 0.7	4.9 ± 1.2	3.5 ± 0.7	2.5 ± 0.9	2.2 ± 0.5	3.0 ± 0.7 **^c^**	1.0 ± 0.2 **^c^**	*p* = .0074	
Center (# of entries)	40.8 ± 7.4	29.3 ± 5.6	32.0 ± 2.8	26.4 ± 5.5	52.8 ± 1.8	42.5 ± 11.1	50.6 ± 10.3	31.2 ± 7.6	31.6 ± 11.0	23.8 ± 0.4	28.3 ± 5.8	17.7 ± 4.6	NS	
Perimeter (% time)	23.5 ± 5.0	39.6 ± 6.5	18.3 ± 1.6 **^a^**	36.9 ± 4.8 **^a^**	21.8 ± 5.8	26.9 ± 6.2	24.3 ± 9.2	36.6 ± 2.5	25.8 ± 8.5	32.2 ± 8.8	18.7 ± 3.5 **^b^**	42.8 ± 6.2 **^b^**	*p* = .0002	
Perimeter (# of entries)	71.2 ± 9.7	92.5 ± 12.9	56.0 ± 6.4 **^a^**	86.3 ± 7.5 **^a^**	55.5 ± 15.4	61.3 ± 4.1	47.4 ± 13.2	71.3 ± 12.6	54.2 ± 8.5	52.3 ± 8.1	62.4 ± 8.7	62.0 ± 6.2	*p* = .0237	
EPM														
Center area (% time)	9.4 ± 1.9 **^d^**	22.9 ± 4.6 **^d^**	10.8 ± 1.1 **^b^**	21.2 ± 3.6 **^b^**	13.2 ± 1.7	10.3 ± 1.5	11.6 ± 2.2	10.8 ± 2.2	7.1 ± 1.2 **^a^**	16.7 ± 4.7 **^a^**	8.5 ± 1.7 **^b^**	18.8 ± 2.4 **^b^**	*p* = .0001	
OTT arm entries (ratio)	0.4 ± 0.1	0.5 ± 0.1	0.3 ± 0.0 **^d^**	0.6 ± 0.1 **^d^**	0.4 ± 0.0	0.5 ± 0.1	0.3 ± 0.0	0.4 ± 0.1	0.5 ± 0.1	0.4 ± 0.1	0.4 ± 0.0	0.4 ± 0.1	*p* = .0047	
OTT arm time (ratio)	0.3 ± 0.1 **^a^**	0.6 ± 0.1 **^a^**	0.2 ± 0.0 **^d^**	0.7 ± 0.1 **^d^**	0.4 ± 0.1	0.5 ± 0.1	0.4 ± 0.1	0.5 ± 0.1	0.6 ± 0.1	0.5 ± 0.1	0.3 ± 0.0	0.5 ± 0.1	*p* = .0016	
Closed arms (# of entries)	10.0 ± 0.6	8.8 ± 1.0	12.2 ± 2.3	7.4 ± 1.6	13.5 ± 1.4	11.5 ± 1.8	12.2 ± 1.9	10.3 ± 0.7	10.7 ± 2.7	12.0 ± 0.8	14.0 ± 1.4	12.0 ± 2.1	NS	
Closed arms (% time)	58.7 ± 8.5 **^a^**	34.0 ± 8.0 **^a^**	68.2 ± 3.4 **^d^**	24.8 ± 5.7 **^d^**	49.9 ± 5.8	41.0 ± 7.4	56.2 ± 10.1	44.8 ± 9.2	37.1 ± 9.1	41.4 ± 7.5	60.5 ± 2.7 **^c^**	40.7 ± 7.3 **^c^**	*p* = .0001	
SPDB														
Burying (frequency)	33.3 ± 11.3	25.2 ± 6.8	20.5 ± 11.1	9.7 ± 3.2	0.6 ± 0.4	7.0 ± 3.5	24.4 ± 10.9	14.2 ± 7.2	3.0 ± 1.1	21.2 ± 7.5	22.1 ± 8.3	10.3 ± 5.4	NS	
Immobility (frequency)	14.5 ± 4.5	12.7 ± 5.8	13.2 ± 3.5	17.7 ± 4.8	21.3 ± 1.6	24.7 ± 5.1	20.8 ± 2.9	19.5 ± 4.8	10.3 ± 4.3	10.8 ± 4.5	8.3 ± 2.6	9.8 ± 4.0	NS	
Rearing (frequency)	9.3 ± 2.4	19.3 ± 4.8	11.0 ± 3.1	20.6 ± 4.3	6.2 ± 2.9	14.8 ± 3.9	12.8 ± 4.3	13.5 ± 5.5	18.7 ± 5.4	15.8 ± 4.0	18.3 ± 2.9	17.7 ± 4.0	NS	
Rearing (% time)	4.5 ± 0.8	11.1 ± 3.7	8.8 ± 3.0	14.5 ± 4.5	6.3 ± 3.1	7.9 ± 2.2	5.7 ± 2.1	6.4 ± 2.3	10.6 ± 3.7	8.8 ± 2.9	9.1 ± 1.8	10.3 ± 2.9	NS	
Probe explore (frequency)	4.8 ± 1.8	14.3 ± 4.4	10.2 ± 4.5	13.4 ± 3.1	6.3 ± 3.1	13.3 ± 4.9	8.8 ± 3.6	9.8 ± 3.0	9.5 ± 3.6	8.2 ± 2.3	13.0 ± 2.9	9.2 ± 2.1	NS	
Probe explore (% time)	1.2 ± 0.2	7.7 ± 3.3	3.9 ± 2.2	9.7 ± 3.3	0.7 ± 0.5	5.3 ± 1.8	3.5 ± 1.7	2.4 ± 0.8	3.3 ± 1.6	4.3 ± 1.6	8.2 ± 3.2	5.1 ± 1.8	NS	
Grooming (frequency)	0.0 ± 0.0	0.0 ± 0.0	0.0 ± 0.0	0.4 ± 0.3	0.2 ± 0.2	0.5 ± 0.3	0.2 ± 0.2	0.5 ± 0.3	0.0 ± 0.0	0.0 ± 0.0	0.1 ± 0.1	0.2 ± 0.2	NS	
Grooming (% time)	0.0 ± 0.0	0.0 ± 0.0	0.0 ± 0.0	0.5 ± 0.3	0.4 ± 0.4	0.7 ± 0.6	0.1 ± 0.1	0.5 ± 0.3	0.0 ± 0.0	0.0 ± 0.0	0.1 ± 0.1	0.1 ± 0.1	NS	
Shock reactivity	1.3 ± 0.2	1.7 ± 0.2	1.5 ± 0.2	1.1 ± 0.1 **^e^**	1.3 ± 0.2	1.0 ± 0.0 **^e^**	1.0 ± 0.0	1.0 ± 0.0 **^e^**	1.5 ± 0.2	1.3 ± 0.2	1.1 ± 0.1	1.0 ± 0.0 **^e^**	NS	
Bedding height (inches)	7.8 ± 0.8	7.8 ± 0.7	6.3 ± 0.8	6.3 ± 0.6	6.1 ± 0.9	5.9 ± 0.4	7.1 ± 1.0	6.8 ± 0.6	5.5 ± 0.4	6.4 ± 0.6	5.8 ± 0.4	6.3 ± 0.5	NS	
NOR														
T1 Avg. Explore (% time)	23.4 ± 2.6	19.6 ± 4.6	24.7 ± 1.7	20.9 ± 1.2	16.0 ± 2.8	22.0 ± 1.6	24.3 ± 3.9	24.6 ± 2.5	N/A	N/A	N/A	N/A	NS	
T1 Discrimination Index	−0.04 ± 0.1	−0.1 ± 0.1	−0.1 ± 0.1	−0.04 ± 0.2	−0.1 ± 0.1	−0.01 ± 0.1	0.1 ± 0.1	0.1 ± 0.0	N/A	N/A	N/A	N/A	NS	
T2 Discrimination Index	0.2 ± 0.0	0.01 ± 0.1	0.1 ± 0.1	0.2 ± 0.0	0.3 ± 0.1	0.2 ± 0.1	0.2 ± 0.1	0.2 ± 0.1	N/A	N/A	N/A	N/A	NS	

Note: EDC, endocrine disrupting chemicals; BPA, bisphenol A; DEHP, di-(2-ethylhexyl) phthalate; OFT, open field test; EPM, elevated plus maze; OTT, open-to-total; SPDB, shock probe defensive burying; NOR, novel object recognition test; T1, training trial; T2, test trial; NS, non-significant; SPDB, shock reactivity was measured on a scale of one to four, ranging from mild 1) to extreme 4) reactivity. Data are presented as mean ± SEM.

^**e**^ *p* < 0.01, difference between control and EDC, females, one-way ANOVA, followed by Fisher’s LSD, *post hoc* analyses.

^**a**^ *p* < 0.05.

^**b**^ *p* < 0.01.

^**d**^ *p* < 0.001, difference between males and females of the same treatment group, two-way ANOVA, followed by Fisher’s LSD, *post hoc* analyses.

^**c**^ *p* = 0.05, difference between males and females of the same treatment group, two-way ANOVA, followed by Fisher’s LSD, *post hoc* analyses.

Figures 2A–D and Table 1 depict the results from the OFT. There was a significant effect of EDC exposure (F (5, 30) = 3.116, *p* = 0.0221) in the time spent in the center zone (%, mean ± SEM); however, females in the BPA (*p* = 0.0633) and B + D (LD) (*p* = 0.05) groups only spent a little more time in the center zone compared to control (Figure 2A), possibly suggesting that they had less anxiety than controls. On the other hand, control (*p* = 0.0327) and B + D (HD) (*p* = 0.0518) females spent less time in the center zone compared to their male counterparts, possibly indicating that these females are more anxiety-prone than the corresponding males (Table 1). Additionally, DEHP (LD) (*p* = 0.0227), DEHP (HD) (*p* = 0.0042), and B + D (HD) (*p* = 0.0255) females had fewer entries into the perimeter zone than control females (F (5, 31) = 2.997, *p* = 0.0262) (Figure 2C), suggesting a possible reduction in anxiety levels in these females. A main effect of sex was additionally determined in perimeter zone entries (F (1, 61) = 5.4, *p* = 0.0237), with significant differences found only within the BPA group (*p* = 0.0281) (Table 1). Finally, there was no effect of EDC exposure in the amount of time males (F (5, 29) = 0.2649, *p* = 0.9286) or females (F (5, 31) = 0.8330, *p* = 0.5363) spent in the perimeter zone. However, a main effect of sex (F (1, 60) = 16, *p* = 0.0002) was observed in this measure (Table 1). BPA (*p* = 0.0267) and B + D (HD) (*p* = 0.0063) females spent more time in the periphery (118.3% and 128.6% more, respectively) compared to their male counterparts, indicating higher levels of anxiety.

### 3.2 Elevated plus maze

**FIGURE 3:**
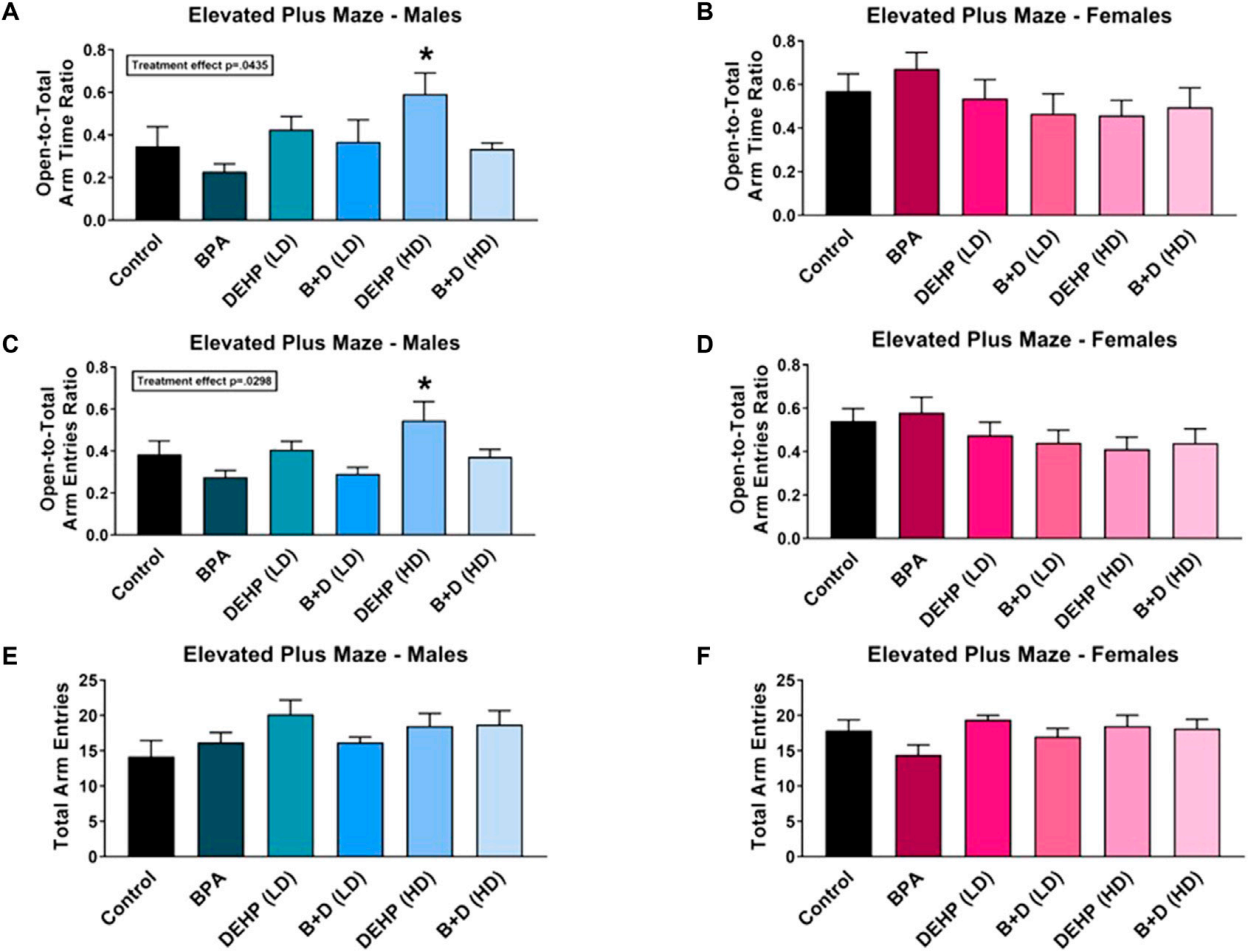
Behavioral effects of prenatal exposure to vehicle or EDCs in adult male and female rat offspring in the elevated plus maze (EPM). **(A)** Time spent in open arms relative to total arms in male offspring, **(B)** time spent in open arms relative to total arms in female offspring, **(C)** number of entries into open arms relative to total arms in male offspring, **(D)** number of entries into open arms relative to total arms in female offspring, **(E)** total exploration of the maze in male offspring, and **(F)** total exploration of the maze in female offspring. Data were collected from adult male and female offspring prenatally exposed to vehicle (Control) (males: *n* = 6; females: *n* = 6), BPA (males: *n* = 5–6; females: *n* = 7), DEHP (LD) (males: *n* = 6; females: *n* = 5–6), DEHP (HD) (males: *n* = 6; females: *n* = 6), a mixture of BPA + DEHP (LD) (males: *n* = 4–5; females: *n* = 6), or a mixture of BPA + DEHP (HD) (males: *n* = 7; females: *n* = 6). Data were analyzed by one-way ANOVA, followed by Fisher’s LSD *post hoc* test. ^*^ *p* < 0.05, comparison between control and EDC-exposed male offspring. Error bars represent the standard error of the mean (SEM).

We did not observe any significant effects of EDC exposure on exploration in the EPM in either sex (Figures 3E,F). However, a main effect of sex (F (1, 59) = 17, *p* = 0.0001) was discovered in the amount of time spent in the central platform of the EPM (Table 1). Furthermore, an interaction effect between EDC exposure and sex (F (5, 59) = 3.0, *p* = 0.0190) was also determined in the central platform time. Females in a majority of the groups spent significantly more time in the center. Control (*p* = 0.0010), BPA (*p* = 0.0069), DEHP (HD) (*p* = 0.0217), and B + D (HD) (*p* = 0.0079) females exhibited robust increases (142.4%, 96.5%, 134.3%, 120.0%, respectively) in center time than their male counterparts, which may be interpreted as increased risk assessment of the maze.

Interaction effects were observed in both open-to-total (OTT) arm time ratio (F (5, 60) = 2.9, *p* = 0.0221) and OTT arm entries ratio (F (5, 59) = 3.0, *p* = 0.0188). DEHP (HD) male offspring had increased ratios (mean ± SEM) of OTT arm time (0.6 ± 0.1) (Figure 3A) and OTT arm entries (0.5 ± 0.1) (Figure 3C) compared to control males (OTT time: 0.3 ± 0.1; OTT entries: 0.4 ± 0.1), suggesting that DEHP (HD) males had reduced anxiety. A similar effect was observed in control (*p* = 0.0477) and BPA (*p* = 0.0002) females, with them having considerably higher ratios for OTT arm time (64.3% and 195.2%, respectively) compared to their male counterparts. BPA females also demonstrated a higher ratio of OTT arm entries than BPA males (females: 0.6 ± 0.1; males: 0.3 ± 0.0; *p* = 0.0007). More interestingly, the enhanced OTT arm time and entries in DEHP (HD) males resembled that in control females (OTT time: 0.6 ± 0.1; OTT entries: 0.5 ± 0.1), suggesting a possible feminization of behavior in these males.

An interaction effect was further observed in closed arm time (F (5, 60) = 2.4, *p* = 0.0444). The corollary of our OTT arm time findings was true in the time spent in the closed arms, with females in the control (*p* = 0.0197) and BPA (*p* = 0.0001) groups spending less time compared to the males (Table 1), confirming reduced anxiety in these two groups. Additionally, B + D (HD) females also spent less time in the closed arms (40.7 ± 7.3; *p* = 0.0506) than their male counterparts (60.5 ± 2.7) (Table 1).

### 3.3 Shock probe defensive burying

**FIGURE 4:**
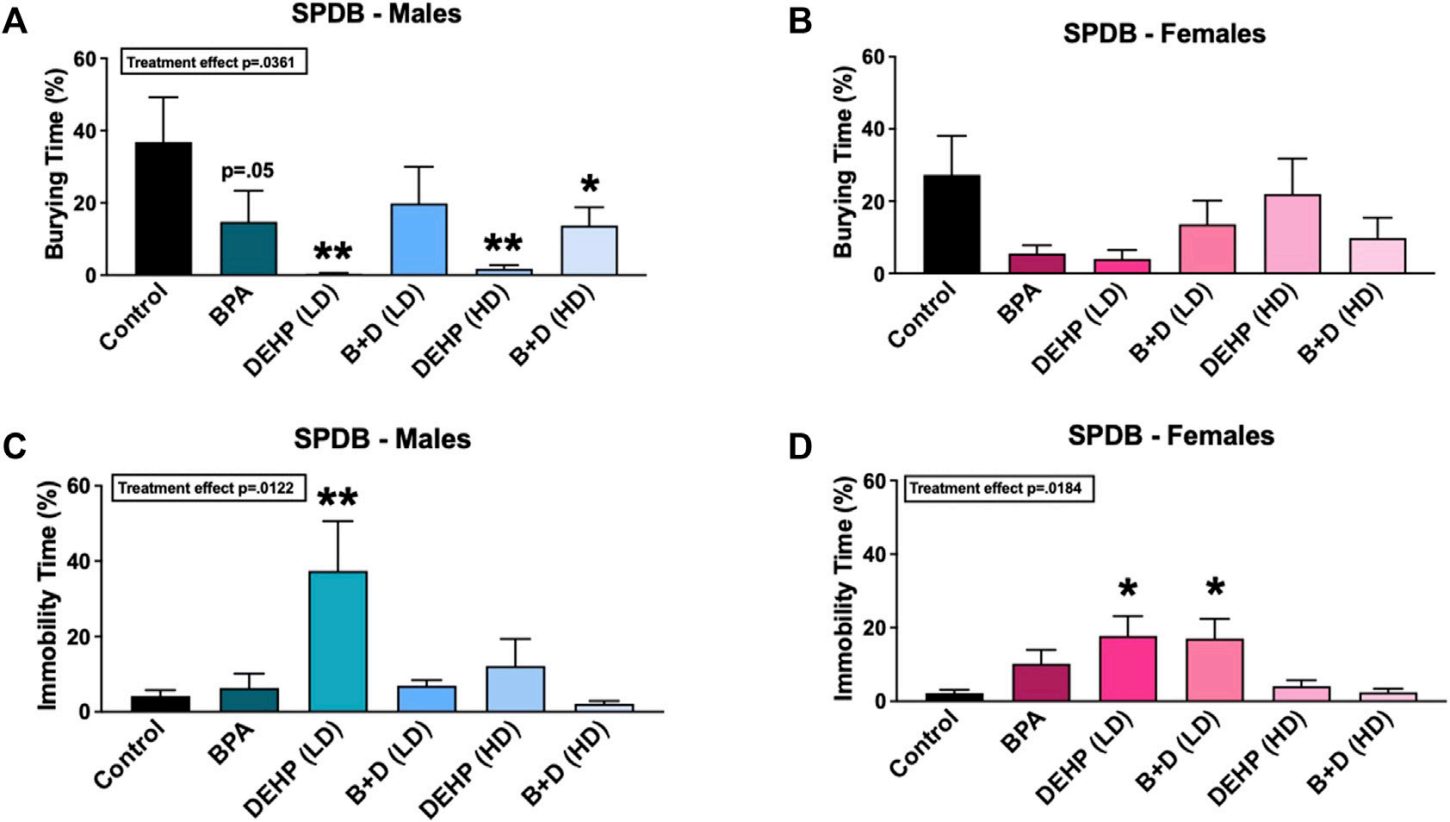
Behavioral effects of prenatal exposure to vehicle or EDCs in adult male and female rat offspring in the shock probe defensive burying (SPDB) test. **(A)** Amount of time spent burying in male offspring, **(B)** amount of time spent burying in female offspring, **(C)** amount of time spent immobile in male offspring, and **(D)** amount of time spent immobile in female offspring. Data were collected from adult male and female offspring prenatally exposed to vehicle (Control) (males: *n* = 5–6; females: *n* = 5–6), BPA (males: *n* = 5–6; females: *n* = 7), DEHP (LD) (males: *n* = 5–6; females: *n* = 6), DEHP (HD) (males: *n* = 5–6; females: *n* = 5–6), a mixture of BPA + DEHP (LD) (males: *n* = 4–5; females: *n* = 6), or a mixture of BPA + DEHP (HD) (males: *n* = 6–7; females: *n* = 5–6). Data were analyzed by one-way ANOVA, followed by Fisher’s LSD *post hoc* test. ^*^ *p* < 0.05, ^**^ *p* < 0.01, comparison between control and EDC-exposed offspring. Error bars represent the standard error of the mean (SEM).

Figure 4 A-D display the results from the SPDB test. A majority of the EDC-treated male offspring demonstrated robust reductions in the amount of time spent burying (%, mean ± SEM) (F (5, 28) = 2.793, *p* = 0.0361) (Figure 4A). Compared to control males (36.9 ± 12.4), males prenatally exposed to BPA (14.8 ± 8.5; *p* = 0.0518), DEHP (LD) (0.3 ± 0.3; *p* = 0.0034), DEHP (HD) (1.8 ± 1.0; *p* = 0.0046), and B + D (HD) (13.7 ± 5.1; *p* = 0.0353) spent significantly less time burying the probe, which indicates a decrease in active coping. DEHP (LD) males (37.4 ± 13.2; *p* = 0.0027) also spent substantially more time immobile than control males (4.2 ± 1.6) (Figure 4C), which is suggestive of passive coping (Fucich et al., 2018). Similar to DEHP (LD) males, DEHP (LD) (*p* = 0.01) and B + D (LD) (*p* = 0.0134) females had over 600% increases in immobility time compared to control females (Figure 4D), indicating that they were prone to passive coping as well. No other differences were observed in burying or immobility frequency, rearing frequency or time, probe exploration frequency or time, or grooming frequency or time in male or female offspring (Table 1).

In addition to these behavioral parameters, a significant EDC effect was observed in female shock reactivity (mean ± SEM) (F (5, 31) = 3.786, *p* = 0.0086) (Table 1). Females prenatally treated with BPA (1.1 ± 0.1; *p* = 0.0089), DEHP (LD) (1.0 ± 0.0; *p* = 0.0018), B + D (LD) (1.0 ± 0.0; *p* = 0.0018), and B + D (HD) (1.0 ± 0.0; *p* = 0.0018) displayed moderately decreased shock reactivity in comparison with control females (1.7 ± 0.2), suggesting possible reduction in fear responses. No such differences were observed in males, nor were there differences in bedding height after EDC exposure in both male and female offspring (Table 1).

### 3.4 Novel object recognition test

**FIGURE 5:**
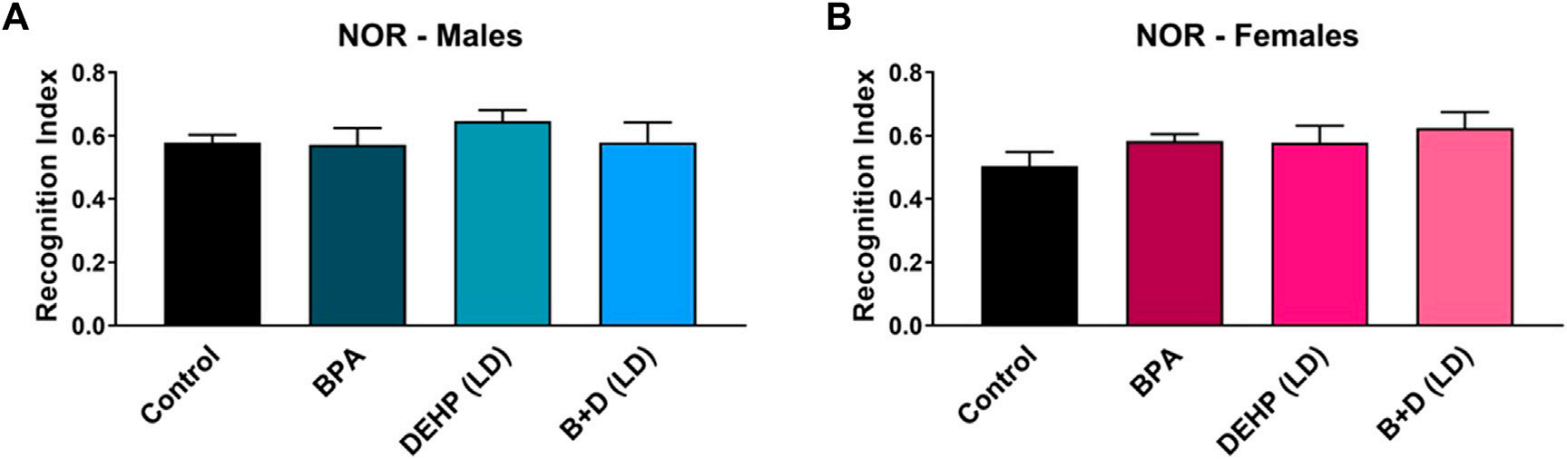
Behavioral effects of prenatal exposure to vehicle or EDCs in adult male and female rat offspring in the novel object recognition (NOR) test. **(A)** Object recognition in male offspring and **(B)** object recognition in female offspring. Data were collected from adult male and female offspring prenatally exposed to vehicle (Control) (males: *n* = 5; females: *n* = 6), BPA (males: *n* = 6; females: *n* = 5), DEHP (LD) (males: *n* = 6; females: *n* = 6), or a mixture of BPA + DEHP (LD) (males: *n* = 5; females: *n* = 5). Data were analyzed by one-way ANOVA, followed by Fisher’s LSD *post hoc* test. Error bars represent the standard error of the mean (SEM).

The results from the NOR are displayed in Figure 5 and Table 1. Two rats were excluded in the EDC-treated groups due to the rats climbing out of the testing box in one or both trials. No significant differences were observed in the EDC-exposed offspring in any of the NOR measures.

### 3.5 Corticosterone levels

**FIGURE 6:**
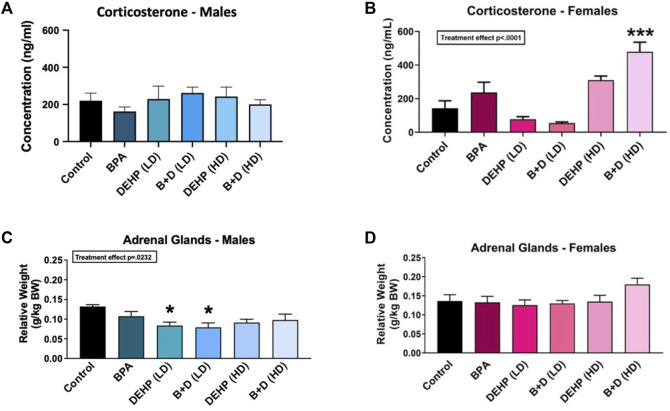
Hypothalamic-pituitary-adrenal (HPA) axis activity in adult male and female offspring prenatally exposed to EDCs or mixtures. **(A)** Serum corticosterone (CORT) levels (ng/mL) measured in male offspring, **(B)** serum CORT levels (ng/mL) measured in female offspring, **(C)** adrenal gland relative weights (g/kg BW) in male offspring, and **(D)** adrenal gland relative weights (g/kg BW) in female offspring. Data were collected from adult male and female offspring prenatally exposed to vehicle (Control) (males: *n* = 6; females: *n* = 6), BPA (males: *n* = 6; females: *n* = 7), DEHP (LD) (males: *n* = 6; females: *n* = 6), DEHP (HD) (males: *n* = 6; females: *n* = 6), a mixture of BPA + DEHP (LD) (males: *n* = 5; females: *n* = 6), or a mixture of BPA + DEHP (HD) (males: *n* = 7; females: *n* = 6). Data were analyzed by one-way ANOVA, followed by Tukey’s multiple comparisons test. ^*^ *p* < 0.05, ^***^ *p* < 0.001, comparison between control and EDC-exposed male offspring. Error bars represent the standard error of the mean (SEM).

An interaction effect was observed in CORT levels (F (5, 35) = 8.427, *p* < 0.0001). BPA in combination with DEHP (HD) exposure significantly increased CORT levels in females compared to controls (*p* = 0.0006) (Figure 6B). However, male offspring did not demonstrate any alterations in CORT as a result of EDC exposure. Sex differences were further observed in DEHP (LD) (*p* = 0.0189), B + D (LD) (*p* = 0.0046), and B + D (HD) (*p* = 0.0008) offspring. In the LD groups, male offspring had significantly higher CORT concentrations than their female counterparts, but this sex difference was reversed in the B + D (HD) group. No further changes were observed in CORT.

### 3.6 Body and organ weights

There was a significant EDC exposure effect (F (5, 30) = 3.082, *p* = 0.0232) in adrenal gland relative weights, with DEHP (LD) (*p* = 0.0362) and B + D (LD) (*p* = 0.0267) male offspring having lower weights than corresponding controls (Figure 6C). In contrast, no significant EDC effects were found in female adrenal gland weights (Figure 6D). Furthermore, there were no differences in male or female body weights (BWs) or pituitary gland relative weights due to EDC treatment (Table 2). However, males had higher body weights and lower pituitary gland relative weights, compared to the female offspring. Moreover, females exposed to both low and high doses of DEHP (*p* = 0.0269 and *p* = 0.0234, respectively) and B + D (*p* = 0.0114 and *p* < 0.0001, respectively) had significantly higher adrenal weights than their male counterparts.

**TABLE 2:** Sex differences in body and organ weights of male and female offspring following low-dose (5 µg) and high-dose (7.5 mg) prenatal EDC exposure.

Parameter	Control		BPA (5 µg)		DEHP (5 µg)		BPA ^a^5 µg DEHP		DEHP (7.5 mg)		BPA ^a^7.5 mg DEHP		Sex effect	
	Male	Female	Male	Female	Male	Female	Male	Female	Male	Female	Male	Female		
Corticosterone (ng/mL)	247.9 ± 45.1	142.6 ± 44.7	182.4 ± 27.2	236.3 ± 61.6	257.9 ± 78.3 **^a^**	77.8 ± 14.8 **^a^**	294.5 ± 35.7 **^b^**	54.2 ± 7.2 **^b^**	273.0 ± 56.8	310.3 ± 24.2	225.5 ± 27.4^**+++**^	478.6 ± 57.6^**+++**^	NS	
Body weights g)	436.2 ± 13.8 **^c^**	290.0 ± 4.6 **^c^**	449.0 ± 7.7 **^c^**	288.7 ± 2.6 **^c^**	462.8 ± 12.2 **^c^**	295.2 ± 4.2 **^c^**	432.4 ± 17.1 **^c^**	274.5 ± 8.8 **^c^**	437.8 ± 11.5 **^c^**	288.0 ± 4.8 **^c^**	446.3 ± 4.3 **^c^**	289.5 ± 9.6 **^c^**	*p* < .0001	
Pituitary relative weights (g/kg BW)	0.03 ± 0.00 **^c^**	0.05 ± 0.00 **^c^**	0.03 ± 0.00 **^c^**	0.05 ± 0.00 **^c^**	0.03 ± 0.00 **^c^**	0.06 ± 0.00 **^c^**	0.03 ± 0.00 **^c^**	0.06 ± 0.00 **^c^**	0.03 ± 0.00 **^c^**	0.06 ± 0.00 **^c^**	0.03 ± 0.00 **^c^**	0.06 ± 0.01 **^c^**	*p* < .0001	
Adrenal gland relative weights (g/kg BW)	0.13 ± 0.01	0.14 ± 0.02	0.11 ± 0.01	0.13 ± 0.02	0.08 ± 0.01 **^a^**	0.13 ± 0.01 **^a^**	0.08 ± 0.01 **^a^**	0.13 ± 0.01 **^a^**	0.09 ± 0.01 **^a^**	0.14 ± 0.02 **^a^**	0.10 ± 0.02 **^c^**	0.18 ± 0.02 **^c^**	*p* < .0001	

Note: EDC, endocrine disrupting chemicals; BPA, bisphenol A; DEHP, di-(2-ethylhexyl) phthalate; BW, body weight. Data are presented as mean ± SEM.

^**a**^ *p* < 0.05.

^**b**^ *p* < 0.01.

^**+++**^ *p* < 0.001.

^**c**^ *p* < 0.0001, difference between males and females of the same treatment group, two-way ANOVA, followed by Fisher’s LSD, *post hoc* analyses.

## 4 Discussion

In this study, we aimed to determine sex differences in the adverse behavioral and cognitive effects resulting from prenatal EDC exposure. The results of our study revealed a myriad of effects on stress-related behaviors, CORT, and adrenal gland weights. Firstly, BPA-treated female offspring displayed a near-significant increase in time spent in the center zone of the OFT, indicative of decreased anxiety-like behavior. Each of the behavioral paradigms incorporated in our study evaluates distinct aspects of stress-related behaviors, with the OFT assessing exploration of a novel environment (Choleris et al., 2001; Ramos, 2008). Hence, our BPA female offspring showed anxiolytic effects in response to novelty. BPA males, on the other hand, spent less time burying in the SPDB. Burying is an adaptive coping style since it embodies an active coping behavioral response to stress (Hori et al., 2004; Bondi et al., 2007). A decrease in burying represents a maladaptive reduction in active coping mechanisms (Fucich and Morilak, 2018). The BPA females in our study also exhibited altered defensive behaviors, but in the form of reduced shock reactivity in the SPDB. These effects taken together suggest that prenatal exposure to BPA at a low dose of 5 µg may lead to aberrant effects on sex-specific defensive behaviors and fear responses.

Prenatal exposure to DEHP at low and high doses yielded varying effects in each of the behavioral tests. In the OFT, both LD and HD DEHP-treated female offspring had fewer entries into the perimeter zone, but showed no changes in perimeter zone time. While a reduction of entries into the perimeter zone of the OFT may represent an anxiolytic-like effect due to reduced activity in the periphery, changes in exploration of the center zone in this test are typically used to measure anxiety-like behavior (Seibenhener and Wooten, 2015; Scholl et al., 2019). DEHP females were unaffected in the center zone parameters, rendering unclear anxiety-related effects.

DEHP (HD) males exhibited decreased anxiety-like behavior in the EPM, which represents a riskier environment and evaluates unconditioned anxiety (Lapiz-Bluhm et al., 2008; Ramos, 2008). This contrasts with studies that have observed anxiogenic effects in the EPM in males with perinatal low-dose (5–40 µg) and high-dose (10–50 mg) DEHP exposure (Xu et al., 2015; Quinnies et al., 2017). These inconsistencies could be attributed to differences in DEHP exposure period, species tested, and age at evaluation. In the aforementioned studies, DEHP was administered to mice throughout the gestational and early postnatal stages, and increased EPM anxiety levels were observed in pubertal male offspring. The anxiolytic effect in our DEHP (HD) males additionally resembled the behavior of control females. The sex difference between HD males and females in the proportion of time spent in the open arms was also reversed compared to controls, and this particular effect was not noted in any other EDC group. This suggests a potential feminization of HD males in anxiety-like behavior. This is consistent with the anti-androgenic effects of DEHP commonly discerned in exposed males (Moore et al., 2001; Vo et al., 2009a; Vo et al., 2009b; Christiansen et al., 2010; Carbone et al., 2013; Wang et al., 2016). We can further confirm based on our results that this particular effect is not present at a lower DEHP dose of 5 µg.

In the SPDB, we observed robust decreases in burying in LD and HD males in the DEHP-exposed groups. LD males and females also engaged in increased immobility, which was not reflected in HD offspring. In addition, we observed significantly reduced shock reactivity in LD, but not HD, females. These findings imply aberrant changes in defensive behaviors–reduced active coping in DEHP males, regardless of dose, and a definite shift to passive coping in DEHP (LD) male and female offspring. An increase in immobility reflects a preference for passive reactive coping mechanisms (Koolhaas et al., 1999; Fucich and Morilak, 2018), a maladaptive stress response that is often observed in individuals with affective disorders, including depression (Koolhaas et al., 1999; Olff et al., 2005; Southwick et al., 2005; Mahmoud et al., 2012). Immobility can also be interpreted as a measure of anxiety-like behavior (Nijssen and Schelvis, 1987).

Heightened immobility responses in the Forced Swim Test, an indicator of depressive-like behavior, have previously been reported in both male and female offspring perinatally treated with DEHP at high doses (10–200 mg/kg) (Xu et al., 2015). Our study is the first to demonstrate an acute stress-induced passive behavioral phenotype in the SPDB in male and female offspring exposed to low-dose DEHP *in utero*. Furthermore, our DEHP (LD) males also showed a reduction in adrenal gland relative weights. This is intriguing because adrenal hormones have been implicated in freezing behavior, with adrenalectomy impairing the duration of fear-induced freezing (Bohus, 1987). Hence, the lower adrenal weights in our DEHP (LD) males may have contributed to their behavioral outcomes. Additional experiments should examine mineralocorticoid and glucocorticoid receptor alterations for further insight.

Treatment with BPA and DEHP in combination altered the behavior of female offspring more than male offspring. B + D (LD) females exhibited anxiolytic effects in the OFT, but passive coping responses in the SPDB. B + D (HD) females showed unclear and slightly conflicting anxiety-like behavioral responses as well. In the OFT, they displayed significantly fewer entries into the perimeter zone, but they also spent the least amount of time in the center zone compared to other female offspring. However, this was not significantly different from control females. Moreover, B + D (HD) females had elevated CORT concentrations relative to their control counterparts. CORT levels were not significantly altered in any of the other treatment groups, regardless of sex. Studies have identified higher CORT levels in female offspring with perinatal BPA exposure at 40 μg/kg (Poimenova et al., 2010; Panagiotidou et al., 2014), but blunted CORT levels in female offspring three generations following prenatal high-dose DEHP exposure (150 mg/kg) (Quinnies et al., 2015). To our knowledge, ours is the first study to report a sex-specific increase in CORT following treatment with a mixture of BPA + DEHP. Interestingly, this outcome was not coupled with any robust behavioral changes.

Similarly, B + D (LD) males were not affected in any of the behavioral measures, but instead had lower adrenal gland relative weights, comparable to DEHP (LD) males. This result is particularly striking because it suggests that B + D (LD) male offspring may be more impacted at the organ systems level. Ours is the first study to our knowledge to determine associations between B + D exposure and reductions in these organ weights in male offspring. B + D (HD) males, on the other hand, did show behavioral alterations. They demonstrated significant reductions in active coping in the SPDB, with no accompanying alterations in CORT or adrenal weights. Therefore, the dose of B + D at which male offspring are exposed to determines the nature of the outcomes they will exhibit.

A major limitation of our study was the fact that the pregnant dams were group housed during the EDC treatment period. As a result, any potential contamination of the animals with bisphenol metabolites released through urine or feces in the cages was not controlled for. However, there were no variations in the types of cages used and materials provided within the cages, and all animals were provided water in glass bottles. Therefore, any additional bisphenol exposure from the environment would have been similar across treatments. Measuring the bisphenol levels in the animals and identifying any differences between EDC-exposed and control animals would help determine if there was bisphenol contamination. Moreover, larger sample sizes in all of our groups may have potentially exposed further significant effects, especially in those parameters that were near-significant.

In addition, all of the female offspring in our study were tested while in the estrus stage of the estrous cycle. This stage is characterized by reduced levels of estradiol (E2) and progesterone P), and is generally correlated with lower stress hormone levels relative to the proestrus stage, during which E2 and P levels are higher (Viau and Meaney, 1991). Anxiety-like behavioral responses are therefore influenced by ovarian steroids such as E2 in females (MohanKumar et al., 2018), but the effects vary based on a multitude of factors, including age. A prior study from our lab reported that chronic exposure to low-dose E2 (20 ng/day for 90 days) increases anxiety-like behavior in female rats (Balasubramanian et al., 2014). Studies have also demonstrated that certain anxiety-related behaviors are independent of estrous cycle stage in female rodents (Scholl et al., 2019). Hence, the fact that only estrus females were used in our study is not a major limitation.

To summarize, offspring with prenatal EDC exposures were dose- and sex-specifically affected in each of the behavioral measures assessed. BPA offspring were impacted in anxiety-like and defensive behaviors. DEHP (LD) offspring were more severely affected in terms of defensive behaviors, whereas DEHP (HD) offspring displayed aberrant anxiety-like behavior. Low dose B + D exposure modified anxiety-like and defensive behaviors, but high-dose exposure affected defensive behaviors and CORT. DEHP and B + D males showed a reduction in adrenal gland weights following low-dose exposure. These findings raise significant health concerns, especially considering the detectable levels of these chemicals in pregnant women’s urine (Arbuckle et al., 2014; Jensen et al., 2016; Muerkoster et al., 2020). Exposure pathways include dermal contact via clothing (Kaylor et al., 2023), food packaging (Pacyga et al., 2019) and the use of personal care products (Fisher et al., 2019). The potential long-term repercussions of these low-dose exposures on offspring behavior warrant attention, emphasizing the need for future rodent studies to delve into molecular mechanisms that influence these behavioral changes. Exploring neurotransmitter systems and hormone receptor dysregulation in the brain could offer valuable insights. Additionally, conducting extensive long-term studies in humans can further elucidate potential behavioral risks.

## Data Availability

The original contributions presented in the study are included in the article, further inquiries can be directed to the corresponding author.
